# A quantitative and non-invasive method for nanoparticle translocation and toxicity evaluation in a human airway barrier model

**DOI:** 10.1016/j.mex.2020.100869

**Published:** 2020-04-21

**Authors:** Fan Zhang, Grace V. Aquino, Erica D. Bruce

**Affiliations:** Department of Environmental Science, Baylor University, One Bear Place #97266, Waco, 76798-7266 TX, United States

**Keywords:** Nanotoxicology, Silver, Lung, Inductively Coupled Plasma Mass Spectrometry (ICP-MS), Transepithelial Electrical Resistance (TEER)

## Abstract

Human exposure to environmental nanoparticles (NPs) may result in systemic distribution and accumulation of NPs. Depending on exposure conditions and their physiochemical properties, NPs could cross biological barriers and reach vital organs. This method describes an analytical technique that quantifies the nanoparticles’ translocation through a sample human airway barrier. Silver nanoparticles (AgNPs) were used as the example nanoparticles due to their common use in nanotechnology. The analytical method introduced in this study allows mass measurements of both cellular uptake and translocation of AgNPs through the modeled barrier. Additionally, cytotoxicity was evaluated using a convenient assay to investigate adverse effects from AgNPs treatment. The assay measures cellular injury from each layer in the barrier independently. The assay does not engage cells physically for chemical reaction, therefore it is non-destructive to the model, and the model can be used for other purposes subsequently. To conclude, this study provides researchers with measurable tools for evaluating the translocation, cellular trafficking, uptake and toxic effects of metallic nanoparticles in the *in vitro* barrier format.

• *Quantitative evaluation of nanoparticles translocation through human airway barrier*
• *Non-invasive and quantifiable toxicity evaluation for co-culture models*

## Graphical abstract

**Figure:**
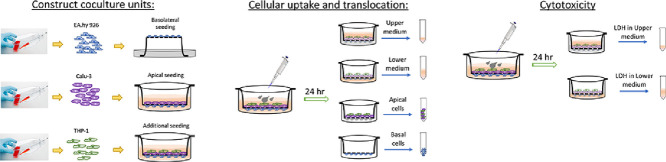

**Specifications Table**

**Table:** 

**Subject Area**	*Select one of the following subject areas:*
	*• Agricultural and biological sciences* *• Biochemistry, genetics and molecular biology* *• Chemical engineering* *• Chemistry* *• Computer science* *• Earth and planetary sciences* *• Energy* *• Engineering* *• Environmental science* *• Immunology and microbiology* *• Materials mcience* *• Mathematics* *• Medicine and dentistry* *• Neuroscience*
	*•****Pharmacology,toxicology andpharmaceutical science***
	*• Physics and astronomy* *• Psychology* *• Social sciences* *• Veterinary science and veterinary medicine*
**More specific subject area:**	*In vitro alternative method, nanotoxicology*
**Method name:**	*Quantitative evaluation of nanoparticles translocation; non-invasive toxicity evaluation in co-culture models*
**Name and reference of original method**	*^1^*Zhang, F., et al. (2015). "Particle uptake efficiency is significantly affected by type of capping agent and cell line." Journal of Applied Toxicology**35**(10): 1114–1121.
**Resource availability**	*Materials and supplies list (Excel file)*

## Method details

### Protocol

**Fig. 1:**
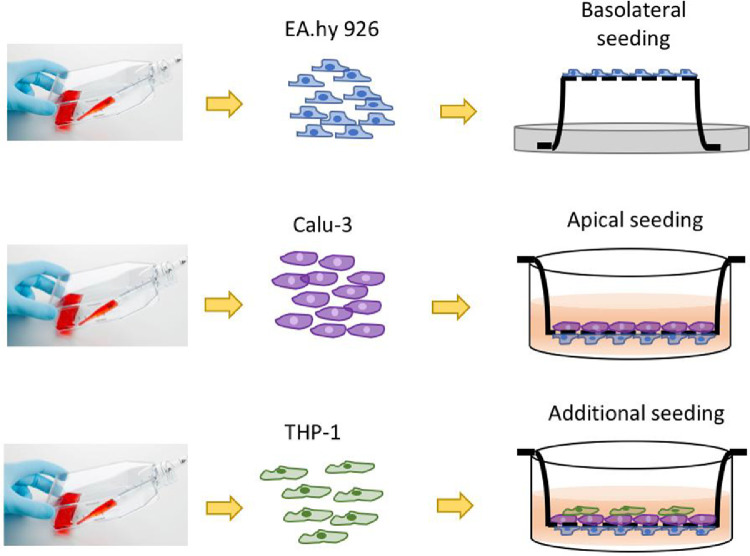
Schematic illustration of coculture barrier fabrication.

**Fig. 2:**
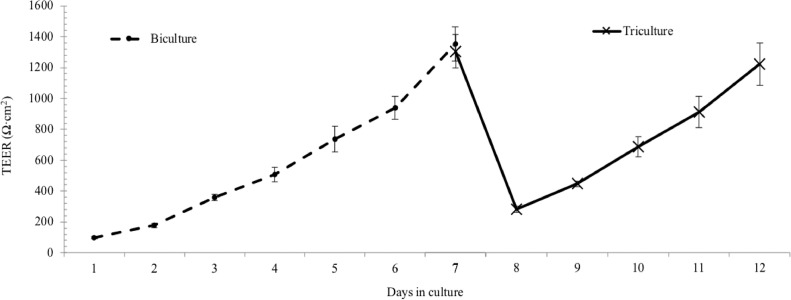
Representative trend of TEER development in the coculture, modified from Zhang et al., 2019. Upon addition of Thp-1 cells and induced differentiation on day 7, a sharp decrease of TEER in the model was observed on day 8. The compromised TEER was slowly restored subsequently, exceeding 1000 Ω•cm^2^ on day 12.

**Fig. 4:**
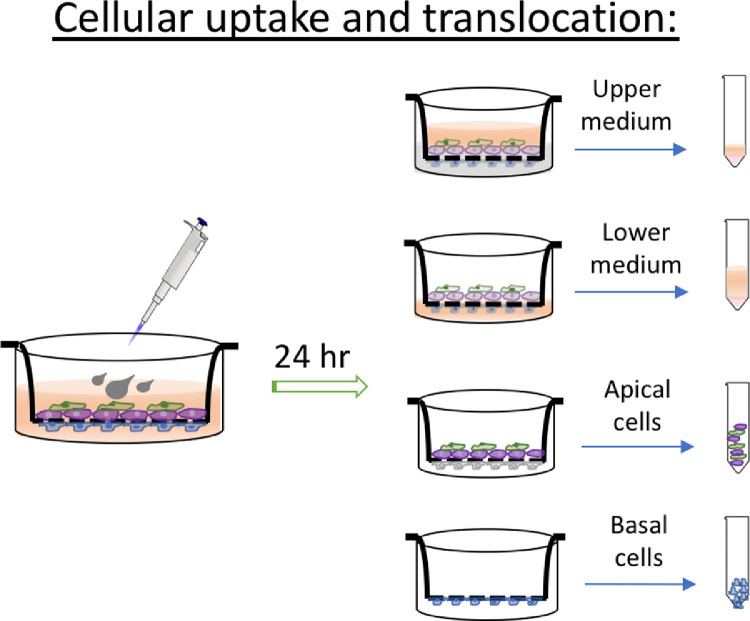
Schematic illustration of sample collection to study cellular uptake and translocation.
  3.2 Collect medium by aspirating from both chambers, and cells grown on apical and basal sides of the inserts. Analyze silver content in each of the following section within the co-culture.
    3.2.1 Collect medium in the insert (upper chamber, about 0.5 ml), sample A.
    3.2.2 Collect medium in the receiving well (lower chamber, about 1.5 ml), sample B.
    3.2.3 Rinse the apical side of insert with 1 mL PBS buffer three times. Collect and combine the rinses with sample A.
    3.2.4 Rinse the basolateral side of the insert and the receiving well with 1 mL PBS buffer three times. Collect and combine the rinses with sample B.
    3.2.5 Add 0.25 and 1 mL of Trypsin-EDTA solution (0.25% Trypsin/0.53 mM EDTA) to the insert and receiving well respectively, and incubate for 3 min at 37 °C to dislodge cells from the membrane insert.
    3.2.6 Carefully collect dislodged cells in apical region of the insert by aspirating, Sample C; and the basolateral region of the insert, Sample D.
    3.2.7 Rinse the membrane insert on both sides with PBS buffer. Collect and combine with their respective cellular samples from the same region.
  3.3 Prepare all samples for quantitative silver content analysis by inductively coupled plasma mass spectrometry (ICP-MS).
    3.3.1 To all samples, add 3 mL of concentrated nitric acid (67–70%), and Rhodium (atomic weight 103) standard solution (1000 μg/mL). Rhodium is used as the surrogate standard for the measurement of silver in this method.
    3.3.2 Apply heat digestion to all samples at 105 °C for 6 h. Add nanopure water to all samples to dilute by a factor of five, resulting in a final Rhodium concentration of 10 mg/L.
    3.3.3 Follow your routine instrument setup and method for metal analysis with ICP-MS. Remember to include 10 mg/L Rhodium to all calibration standard solutions. Prepare all calibration standards in 2% nitric acid with your target analyte (Ag, in this study), and at least five rising concentrations such as 0, 1, 10, 25, 50 and 100 mg/L. It is also suggested to include 2% nitric acid and calibration standard (e.g. 1 and 10 mg/L) samples in between experimental samples as quality control.
4 Cytotoxicity measurements
  4.1 Before AgNPs treatment, collect conditioned medium (spent media harvested from cultured cells) from each sample to serve as control (before treatment). Media in the inset and in the receiving well need to be collected and stored separately.
  4.2 Dose cells accordingly and measure cytotoxicity after 24 h. The cytotoxicity of AgNPs is evaluated by comparing lactate dehydrogenase (LDH) level in the extracellular environment (in culture medium) before and after AgNPs treatment (Fig. 6).
    4.2.1 Prepare LDH assay reagents.
      4.2.1.1 LDH reagents consist of 200 mM TRIS solution (pH 8), 50 mM Li lactate, and 2-p-iodophenyl-3-p-nitrophenyl-5-phenyl tetrazolium chloride (INT)/phenazine methosulfate (PMS) in nicotinamide adenine dinucleotide (NAD) solution. Mixing ratio of TRIS, Li lactate and PMS/INT/NAD is 1:1:1. Every 15 ml of the LDH assay reagents is good for 100 individual tests in a 96-well plate.
      4.2.1.2 Recipes to make above mentioned solutions in bulk are
        4.2.1.2.1 100 ml of 200 mM TRIS - combine 2.222 g Tris–HCl and 1.06 g Tris-base with 100 ml nanopure water,
        4.2.1.2.2 100 ml of 50 mM Li lactate- add 1.96 g lithium lactate to 100 ml nanopure water,
        4.2.1.2.3 10 ml INT- dissolve 330 mg INT in 10 ml DMSO at 33 mg/ml,
        4.2.1.2.4 10 ml PMS- dissolve 90 mg PMS in 10 ml nanopore water at 9 mg/ml,
        4.2.1.2.5 92 mL NAD- dissolve 0.344 g NAD in 92 ml nanopore water at 3.74 mg/mL.
        4.2.1.2.6 INT/PMS/NAD mixture- Mix together 200 μl INT, 200 μl PMS and 4.6 ml NAD for volume of 5 ml. Prepare the mixture fresh before using, as it gradually darkens over time.
      4.2.1.3 Mix together 5 mL of TRIS, 5 mL of Li lactate, and 5 mL of NAD/PMS/INT solution to make complete LDH assay reagents.

1 Cell lines and culture media specification/preparation
  1.1 Obtain the cell lines from a reliable source. The model uses the following cell types: human vascular endothelial cells EA.hy926, human bronchial epithelial cells Calu-3 and human acute monocytic leukemia Thp-1. Maintain the cultures following provider's instruction in proper media under appropriate culture conditions until the desired confluence. Standard culture conditions for above mentioned cells are 37 °C, humid air mixture containing 5% CO_2_.
  1.2 The three selected cell types in the co-culture model each require its own type of media. Prepare and store all media in sterile conditions, and use them in biological safety cabinets only. Product details (such as vendor and catalog number) are summarized in the material list spreadsheet in the supporting information.
    1.2.1 All media are prepared with 10% fetal bovine serum (FBS) and 1% penicillin-streptomycin (PS) supplement. Store FBS and PS routinely in −20 °C. Before use, thaw heat-inactivated FBS and PS aliquots in water bath at 37 °C.
    1.2.2 Make medium for endothelial EA.hy926 cells.
      1.2.2.1 First combine Dulbecco's Modified Eagle's Medium (DMEM) powder formula, 1.5 g of sodium bicarbonate, 0.11 g sodium pyruvate, 4.5 g d-glucose, 2 vials of l-glutamine (0.292 g per vial), 15.9 mg phenol red and thawed 10 mL PS with 900 ml nanopure water (18.2 Ω-cm purity) in a 1000 mL graduated cylinder.
      1.2.2.2 Adjust medium pH level to 7.25 with 1 M hydrochloric acid (HCl) or 1 M Sodium hydroxide (NaOH) solutions, before adding 100 mL of thawed FBS.
      1.2.2.3 Stir well, and filter the 1000 mL medium through a 0.2 µm sterile filter into sterile medium container. This medium is referred as DMEM-high, due to its higher glucose level compared to regular DMEM formula.
    1.2.3 Make medium for epithelial Calu-3 cells.
      1.2.3.1 Similarly, combine DMEM powder formula, 1.5 g of sodium bicarbonate, 0.11 g sodium pyruvate, 1.5 g d-glucose, 1 vial of l-glutamine (0.292 g), 15.9 mg phenol red and thawed 10 mL PS with 900 mL nanopure water in a 1000 mL graduated cylinder.
      1.2.3.2 After adjusting medium pH level to 7.25, add 100 mL thawed FBS to the cylinder.
      1.2.3.3 Filter the 1000 mL medium through a 0.2 µm sterile filter after thorough mixing. This medium is referred as DMEM-low because of the relative low glucose level.
    1.2.4 Make medium for monocytic leukemia Thp-1 cells.
      1.2.4.1 Prepare Roswell Park Memorial Institute (RPMI) 1640 medium by combining RPMI 1640 medium powder formula, 1.5 g sodium bicarbonate, 0.11 g sodium pyruvate, 4.5 g d-glucose, 2.383 g 4-(2-hydroxyethyl)−1-piperazineethanesolfonic acid (HEPES), 15.9 mg phenol red and thawed 10 mL PS in 900 mL nanopure water.
      1.2.4.2 Adjust its pH to 7.25 with HCl and NaOH, and supplemented with 100 mL thawed FBS before filtering through a 0.2 µm sterile filter into storage container.
    1.2.5 Store all media at 4 °C, and warm to 37 °C prior to use on cells in sterile environment.
2 Co-culture model
  2.1 The complete air-blood barrier is built upon a mature bi-culture of epithelial and endothelial cells (Fig. 1).
    2.1.1 First, build the bi-culture of EA.hy926 and Calu-3 by growing them on the opposite sides of a 1.0 µm pore size membrane insert. This protocol uses polyester membrane inserts in a 12-well plate format.
      2.1.1.1 Invert the membrane inserts in a petri dish, with the bottom surface facing up. One petri dish (100 × 21 mm) can fit up to 6 inserts.
      2.1.1.2 Carefully seed 200 µl EA.hy926 cells onto the basolateral membrane at a density of 2.5 × 10^4^ cells/cm^2^. Cover these inserts in the petri dish, and cautiously transfer them to the incubator, awaiting cell attachment for 2 hr.
      2.1.1.3 Remove the inserts from incubator and gently aspirate the excess medium from each insert without scratching the attached cells. Rinse the insert surface with fresh medium twice to remove any loosely attached cells.
      2.1.1.4 Flip the membrane inserts and place them in the receiving well in a 12-well plate. Add 1.5 mL of DMEM-high medium to each receiving well to sustain the attached endothelial cells. Cell attachment on the basolateral side of inserts can be confirmed under a light microscope.
      2.1.1.5 Next, add 500 µl Calu-3 cells to the apical side of the insert in the density of 5.0 × 10^4^ cells/cm^2^. Cell attachment takes up to 4 h (attachment ratio is approximately 50%) in DMEM-low medium. Renew cell media from both compartments every other day. Working volumes of medium in receiving well and insert are 1.5 and 0.5 ml respectively for 12-well plates.
      2.1.1.6 The maturity of bi-culture units is monitored daily by measuring transepithelial electrical resistance (TEER). See 2.2 for TEER measurement details.
    2.1.2 When TEER value of a bi-culture unit has exceeded 1000 Ω•cm^2^, introduce Thp-1 monocytic cells into the bi-culture system and induce Thp-1 differentiation to macrophage-like cells in situ.
      2.1.2.1 Remove medium from the apical region of the insert, and replace with DMEM low/RPMI 1640 mixture (mixed media) in the ratio of 5:1, containing 2.0 × 10^4^ Thp-1 cells.
      2.1.2.2 After introducing the cells, add phorbol 12-myristate 13-acetate (PMA) into the apical medium to a final PMA concentration of 100 ng/ml and incubate the cultures at 37 °C for 48 h to allow differentiation of the Thp-1 cells to macrophage-like cells.
      2.1.2.3 At the end of incubation period, discard the PMA-containing medium and replace with fresh mixed media.
    2.1.3 Rest the differentiated Thp-1 cells for another 48 h to reach the optimal differentiation status, and they will attach to the apical surface Calu-3 layer. The differentiation of Thp-1 and attachment to Calu-3 will result in a steep reduction of TEER; however, TEER value will be restored in a few days.
    2.1.4 Monitor TEER development daily until above 1000 Ω cm^2^ again for the final triple-culture barrier.
  2.2 Barrier property confirmation
    2.2.1 Confirm proper barrier property by measuring TEER of the constructed barrier using a Voltohmmeter with STX2 electrodes (Fig. 2). A high TEER value indicates a strong and tight barrier. The bench mark resistance is commonly set at 1000 Ω•cm^2^. [Note: it is advised to check the tissue-specific TEER value *in vivo* to set the bench mark, for models simulating other barriers.]
    2.2.2 First, determine the TEER from a cell-free insert, which is the native resistance from the porous membrane, TEER_0_ (background value).
      2.2.2.1 Place a cell-free insert in a well that contains 1.5 mL of DMEM-high medium, and add 0.5 mL of DMEM-low medium into the apical region.
      2.2.2.2 Sanitize the Voltohmmeter electrodes in 70% of ethanol, and then wash it in culture media before introducing the electrodes into the culture.
      2.2.2.3 Carefully put the Voltohmmeter electrodes into samples in straight upright position. Leave the short electrode in the apical region of insert, and the long electrode outside the insert in the well touching its bottom. Read the resistance value TEER_0_.
    2.2.3 Next, measure the TEER of culture barrier, TEER_e_.
      2.2.3.1 Follow the same procedure for sanitizing and positioning the electrodes. Make sure the long electrode touches the bottom of receiving well, but the short electrode in the insert is away from the tissue layer. This normalizes the electrodes positions from each reading, and will protect the culture from being scratched by the electrodes.
      2.2.3.2 Read the resistance value, TEER_e_, on the Voltohmmeter screen, and use the following formula to determine the calculated electrical resistance of cultured barrier, where A is the surface area of the membrane (1.12 cm^2^ for 12-well plate inserts).
        TEER = (TEER_e_ – TEER_0_) × *A*
      2.2.3.3 Take three TEER measurements for each sample to get a daily average, and compare it to 1000 Ω cm^2^ to determine if additional culturing is needed. When measuring TEER in the culture, it is imperative to act efficiently to reduce artifact, as temperature change affects medium conductivity and thus the resistance reading.
3 Cellular uptake and translocation of NPs
  3.1 In this study, the example NPs are 50 nm (transmission electron microscopy diameter) tannic-coated AgNPs. They have been fully characterized by the manufacturer and in house using several instrumentations on size, shape and surface charge. For detailed characterization of these nanoparticles, please refer to [1]. The cellular uptake and translocation of AgNPs were evaluated in a 24 hr exposure scenario at 37 °C and 3 mg/L dosing concentration (Fig. 4).
    3.1.1 Obtain or synthesize AgNPs in the pure and concentrated sterile form. The AgNPs used in the study are highly concentrated at 1 mg/ml, sterile, endotoxin- and residual reactants- free. Once triple-culture is ready (TEER is restored to 1000 Ω cm^2^), add 1.5 µL of the stock AgNPs to the apical side of the inserts (0.5 mL of mixed media of RPMI and DMEM-low) to final concentration of 3 mg/L.
    3.1.2 Return culture to incubator and maintain at 37 °C for 24 hr.

**Fig. 6:**
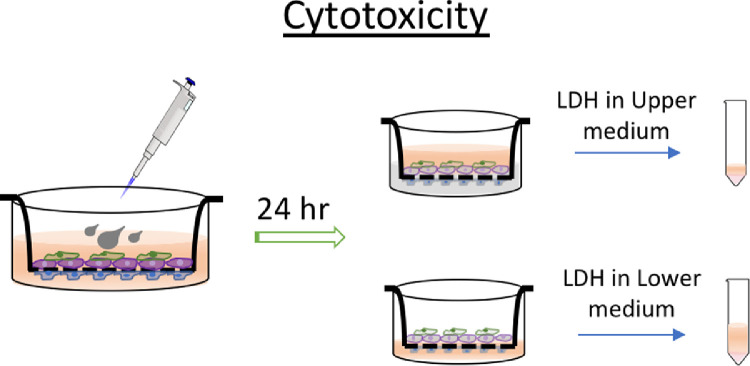
Schematic illustration of sample collection to evaluate cytotoxicity of AgNPs to the model.
  4.3 Upon completion of AgNPs treatment, collect the media in the insert and in the receiving well separately.
  4.4 Combine every 1 ml conditioned medium (control and treated) with 3 ml LDH assay reagents in a mixing tube. Transfer the mixed solution to a clean 96-well plate at 200 μl per well. Include at least 8 replicates per treatment condition. Perform the assay away from light.
  4.5 Wait 5 min. Read the plate for optical absorbance at 490 nm on a microplate reader.
  4.6 Data from control samples (before treatment) indicate spontaneous LDH level which is the baseline for toxicity comparison. The induced LDH from AgNPs treatment is the difference between spontaneous LDH and observed LDH in treated samples. Toxicity is shown by comparing LDH leakage from treatment groups to leakage in control (spontaneous LDH leakage). This is a relative comparison considering the control as the baseline. The exact LDH concentration in the extracellular matrix of LDH, can be determined by fitting the LDH optical absorbance into a pre-established LDH standard curve (optical density curve against LDH concentration). Another alternative is to present LDH leakage in the% max leakage. The max LDH leakage is determined by lysing same amount of cell with 1% of Triton X solution. The% max leakage is then calculated by LDH (observed) / max LDH (cell lysis) * 100%. Finally, apply appropriate statistical test to indicate significant difference in the groups. [Note: each barrier unit contains four sets of data: apical control, basal control, apical treated and basal treated. Additional treated groups will add to the data set if more dosing concentrations are tested.]

## Additional information

Human exposure to environmental nanoparticles (NPs) becomes inevitable due to their wide range of applications in agricultural, industrial and medical fields. Depending on exposure conditions and their physiochemical properties, NPs could cross biological barriers and reach vital organs. Translocation or efflux of nanoparticles through biological barriers has been routinely evaluated by animal models, due to the architectural limitation of conventional *in vitro* models. New cellular models that use three-dimensional co-culture have the potential of providing more physiologically relevant condition and obtaining more predictive data. This paper introduces a method that quantitatively evaluates the translocation and toxicity of NPs in co-culture settings.

The quantification methods were adopted and improved from Zhang et al., 2015 [1]. The original method was proven valuable in the assessment of cellular uptake and toxicity in conventional monoculture models. However, the model has inherent architectural limitation and lacks the power to evaluate efflux of AgNPs in a dynamic mode. The improved coculture model, owing to the membrane insert, has an added space dimension (y axis) and is closer to a physiological barrier. Movement of NPs in both the lateral (x) and vertical (y) directions between chambers become measurable. The adapted analytical methods were applicable to use in the new model and proven useful in the quantification of mass in the dynamic setting. The quantified translocation is particularly valuable information in the assessment of drug delivery system and toxicant distribution systemically.

**Fig. 3:**
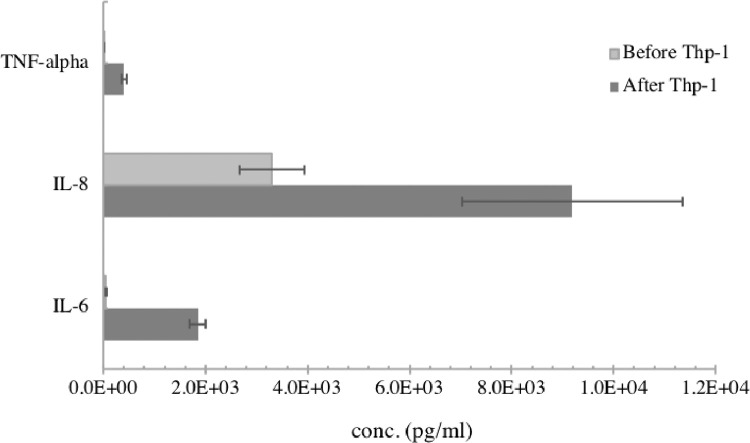
Representative cytokine/chemokine expression upon Thp-1 introduction and differentiation. The change in pro-inflammatory markers expression confirms Thp-1 differentiation, and possibly explains the significant reduction of TEER observed on day 8. Figure was modified from Zhang et al., 2019.

**Fig. 5:**
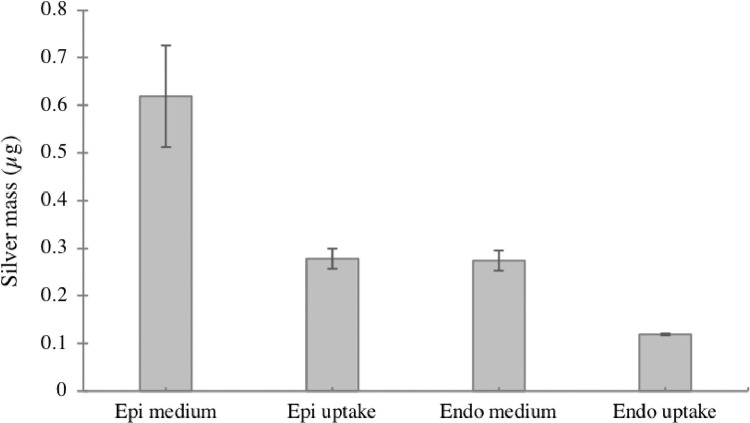
Representative results of cellular uptake and translocation using presented method.

**Fig. 7:**
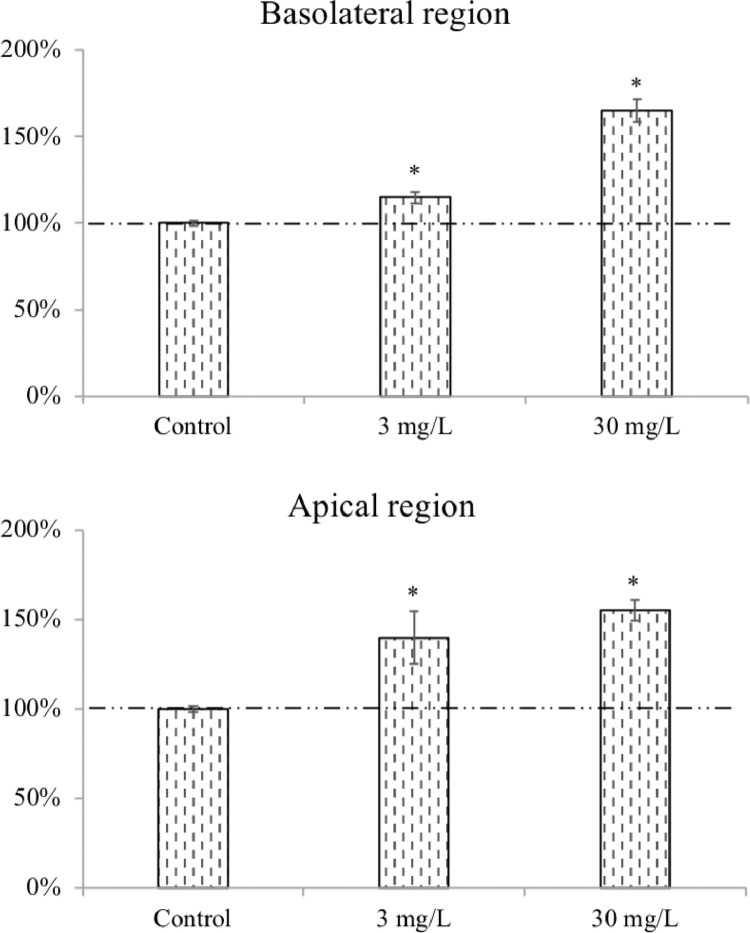
Representative results of LDH measurement in the model after AgNPs treatment. Data from basolateral region indicates injury from endothelial layer (EAhy926 cells), while the apical region represents toxicity from epithelial (calu-3) and macrophage (differentiated Thp-1) mixture. Control showed spontaneous LDH release from cells under native condition, and increased LDH from treatment groups inform cellular injury due to AgNPs exposure.

For the development of the coculture barrier, the protocol details the steps to assemble a sample human airway barrier model (Fig. 1). The model is comprised of three cell types in a two-chamber structure including epithelial, endothelial and macrophage-like cells. The resultant barrier does not claim to be the closest mimic of human air-blood barrier, but rather serves as a template to illustrate the steps in barrier development. The assembly method is highly adaptable. Researchers are encouraged to follow this template and customize barriers with cell types that best suit their research purposes. Additional biological barriers such as the blood-brain, intestinal, placenta barriers can also be simulated using this system. The barrier presented here was characterized for barrier integrity (Fig. 2) and macrophage activation (Fig. 3) in the previously published manuscript. Finally, translocation and toxicity were assessed using the method described herein, and results indicate significant efflux of AgNPs through the barrier (Fig. 5) along with measurable cytotoxicity (Fig. 7) [2].

The presented barrier utilizes a physical membrane to support and separate the cells from opposite sides. One important limitation of incorporating membrane in these models is the retention of tested compound in its pores. As much as 30% of Ag was retained in the membrane as stated in our previous study (Zhang et al., 2019). The same issue was also reported by [3] where fluorescence-labelled nanobeads were absorbed on Transwell® membrane. Therefore, it is imperative to conduct an acellular retention test prior to the actual research to determine retention rate of the tested subject. Membrane inserts are available in various pore sizes. Larger pore membranes are generally more permeable, but they could also be too leaky to support cell growth. Considering features of both permeability and tissue support, we chose the 1 μm pore size for the membrane used in this study.

In this study, the authors used a Voltohmmeter to confirm barrier integrity by measuring TEER. Other methods available that inform barrier properties include immunostaining adherent and tight junction proteins (e.g. E-/VE- cadherin, ZO-1) [4], and permeability measurements of Lucifer Yellow CH dipotassium salt transported from apical to basal side of the barrier [5]. The LDH bioassay may also be substituted by TEER measurement directly in the barrier to indicate toxicity. While a compromised membrane can result in decreased TEER [6], the instrument may not be sensitive enough to detect minor cellular damage. Other viability screening assays, such as tetrazolium-based colorimetric assay (MTT), can be an alternative to LDH measurements, since recently there have been concerns that some metal particles could interfere with LDH reagents and produce false results [7]. However, assays like MTT and other colorimetric or fluorometric assays share one common limitation. These assays interact with cells directly by engaging cells and deprive them in the chemical reaction. The advantages of using LDH assay are noteworthy. It measures analyte in the extracellular matrix and not in cells, so the tissue remains undisturbed and can be reused for other purposes (such as uptake analysis). The reusability of costly coculture models is a valuable feature for either the in-house-made or commercial units.

LDH leakage from treatment groups are compared to the level of spontaneous leakage from the control. This is a relative comparison, considering the control as the baseline. The exact LDH concentration in the extracellular matrix of LDH can be determined by fitting the LDH data onto a pre-established LDH standard curve. Another alternative is to present LDH leakage in the% max leakage. The max LDH leakage is determined by lysing the same amount of cells with 1% of Triton X solution. The% max leakage is then calculated using the sample absorbances in the following formula: LDH (observed) / max LDH (cell lysis) * 100%.

## Conflict of Interest Statement

The authors declare no conflicts of interest.

## Appendix Supplementary materials

Supporting information Image, application 1
